# Intercellular interaction mechanisms promote diversity in intracellular ATP concentration in *Escherichia coli* populations

**DOI:** 10.1038/s41598-022-22189-x

**Published:** 2022-10-26

**Authors:** Ryo J. Nakatani, Masahiro Itabashi, Takahiro G. Yamada, Noriko F. Hiroi, Akira Funahashi

**Affiliations:** 1 Graduate School of Fundamental Science and Technology, Center for Biosciences and Informatics, Keio University, Yokohama, Kanagawa 223-8522 Japan; 2 Present Address: Department of Biosciences and Informatics, Keio University, Yokohama, Kanagawa 223-8522 Japan; 3 School of Medicine, Keio University, Shinjuku-ku, Tokyo 160-8582 Japan; 4 Faculty of Creative Engineering, Kanagawa Institute of Technology, Atsugi, Kanagawa 243-0292 Japan

**Keywords:** Population dynamics, Systems analysis

## Abstract

In fluctuating environments, many microorganisms acquire phenotypic heterogeneity as a survival tactic to increase the likelihood of survival of the overall population. One example of this interindividual heterogeneity is the diversity of ATP concentration among members of *Escherichia coli* populations under glucose deprivation. Despite the importance of such environmentally driven phenotypic heterogeneity, how the differences in intracellular ATP concentration emerge among individual *E. coli* organisms is unknown. In this study, we focused on the mechanism through which individual *E. coli* achieve high intracellular ATP concentrations. First, we measured the ATP retained by *E. coli* over time when cultured at low (0.1 mM) and control (22.2 mM) concentrations of glucose and obtained the chronological change in ATP concentrations. Then, by comparing these chronological change of ATP concentrations and analyzing whether stochastic state transitions, periodic oscillations, cellular age, and intercellular communication—which have been reported as molecular biological mechanisms for generating interindividual heterogeneity—are involved, we showed that the appearance of high ATP-holding individuals observed among *E. coli* can be explained only by intercellular transmission. By performing metabolomic analysis of post-culture medium, we revealed a significant increase in the ATP, especially at low glucose, and that the number of *E. coli* that retain significantly higher ATP can be controlled by adding large amounts of ATP to the medium, even in populations cultured under control glucose concentrations. These results reveal for the first time that ATP-mediated intercellular transmission enables some individuals in *E. coli* populations grown at low glucose to retain large amounts of ATP.

## Introduction

Many species of microorganisms acquire phenotypic heterogeneity to withstand fluctuations in their environments^1–3^. As one type of phenotypic variation, metabolic heterogeneity plays an important role in the survival of microorganisms under stress^4–6^. For example, recent research focusing on the relationship between intracellular ATP concentrations and stress survival^7–10^ has shown that under glucose-deficient conditions, only some of the members of a monoclonal population of *E. coli* acquire high concentrations of ATP^11^. Given that ATP is vital to both metabolism and biochemical reactions, ATP and metabolic heterogeneity can be considered to be inseparable. Against this background, evidence has begun to surface supporting the regulation of aerobic and anaerobic respiration as a survival tactic^4^, but how these differences are controlled in individual cells to produce some that retain high levels of ATP (i.e., the mechanism generating metabolic heterogeneity) remains unclear.

Currently, mechanisms that produce phenotypic heterogeneity are characterized according to four classifications: stochastic state switching, periodic oscillation, cellular age, and intercellular transmission^12^. Stochastic state switching results from unbalanced albeit random gene transcription, biochemical reactions, and distribution of cellular components upon cytokinesis^13–16^. To investigate the involvement of stochastic state transitions in phenotypic heterogeneity, Nakashima et al. proposed a lineage expectation–maximization algorithm that estimates state transitions in a cell lineage by defining each state in individual lineages as a hidden state of Hidden Markov Model^17^. Similar to stochastic state switching, periodic oscillation arises due to a stochastic phase lag in oscillatory biochemical reactions^18,19^. In contrast, phenotypic heterogeneity via cellular age occurs through a rule-based biased partition of protein aggregates or cellular membrane proteins that leads to the concentration of these components in some members of a population^20,21^. A cellular age–type mechanism may be applicable to *E. coli* survival because its cellular membranes are elongated from the center, such that ’old’ cellular poles remain in only some individual organisms. This difference in phenotype also arises between daughter cells^22,23^. For mechanisms of phenotypic heterogeneity via cellular transmission, interindividual cooperation is mediated not only by quorum-sensing molecules transmitted through extracellular diffusion^24,25^ but also through direct intercellular contact by pili and nanotubes to transmit cytosolic components such as plasmids and proteins^26,27^. The influence of intercellular transmission on phenotypic heterogeneity has been addressed by statistical analysis methods that account for both the influence of lineage and of spatial factors on cell lineage^28^. Using information on the spatial arrangement of cells and cell lineage, this method quantifies the influence of lineage factors as the ratio of phenotypic differences between the closest related cell and the cell equidistant from it for each cell; the influence of spatial factors is calculated as the ratio of phenotypic differences between the spatially nearest neighbor and its sister cells. When lineage factors have little to no influence and the influence of spatial factors is significantly higher, intercellular transmission can be judged as generating phenotypic heterogeneity.

Depending on the cell lineage and mechanism involved, all four of the aforementioned molecular mechanisms show distinct differences regarding when phenotypic heterogeneity emerges^12^. For example, heterogeneity due to cellular ageing, with its rule-based biased partitioning, emerges among the first progeny of a population, and the resulting phenotypic differences tend to widen as the population enlarges. In contrast, the development of phenotypic heterogeneity due to cellular transmission is less chronologically consistent than that due to cellular ageing and instead rests on when necessary conditions regarding population density and intermember positioning are achieved. For stochastic state switching and periodic oscillations, phenotypic heterogeneity follows a predetermined ratio but not a defined relationship between the individual and phenotype, because the changes in phenotype are driven by stochastic factors. As these examples show, these different molecular mechanisms demonstrate unique time points at which phenotypic heterogeneity arises. Therefore, chronological monitoring of the emergence of phenotypic heterogeneity may reveal the particular underlying mechanism.

We hypothesized that the diversity in the intercellular ATP concentration between members of *E. coli* populations under glucose deprivation results from one (or more) of the molecular mechanisms that cause phenotypic heterogeneity. By observing the emergence of ATP concentration diversity in single *E. coli* cells under glucose-deficient conditions, we sought to determine which of these mechanisms is used to achieve this survival tactic in *E. coli* populations. To this end, we generated cellular lineages from cells that demonstrated spatiotemporal differences in intracellular ATP concentration. Analysis of these cellular lineages revealed that cellular transmission dependent solely on the spatial relationship among cells is essential in causing ATP diversity among individual cells of *E. coli* populations under glucose-deficient conditions. We then focused on metabolites that might contribute to this metabolic heterogeneity and found that extracellular ATP (eATP) promotes diversity of intracellular ATP concentration in *E. coli* populations.

## Results

### Confirmation of intercellular diversity of intracellular ATP concentration in *E. coli*

**Figure 1:**
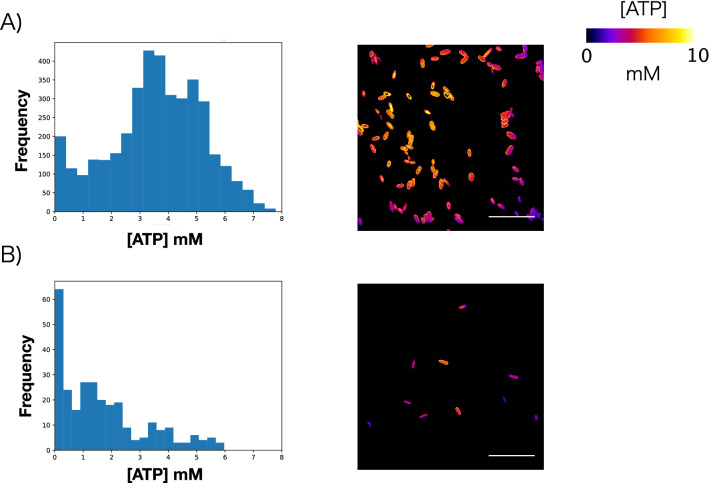
Diversity in intracellular ATP concentration in individual *E. coli* cells under glucose-deficient conditions. (**A**) Histogram of intracellular ATP concentrations of individual *E. coli* cells under control conditions (22.2 mM glucose; skewness = − 0.273, n = 3919) and a microscopic image of color-rendered ATP concentrations in individual cells. (**B**) Histogram of intracellular ATP concentrations of individual *E. coli* under glucose-deficient conditions (0.1 mM glucose; skewness = 0.972, n = 359) and a microscopic image of color-rendered ATP concentrations in individual cells. Scale bar, 10 μm.

To measure intracellular ATP concentrations, we cultured *E. coli* carrying the QUEEN-2m plasmid^11^ under glucose-deficient (0.1 mM glucose, $30\,\,^{\circ }\hbox {C}$) and control (22.2 mM glucose, $30\,\,^{\circ }\hbox {C}$) conditions. The QUEEN-2m protein contains GFP as a fluorescent subunit. The binding (or non-binding) of ATP changes the conformation of a truncated protein $\epsilon$ from *B. subtilis* in the QUEEN-2m and thus alters the excitation spectrum. The QUEEN-2m spectrum has peaks at excitation wavelengths of 400 nm and 494 nm; ATP binding decreases the GFP fluorescence for the excitation wavelength of 494 nm relative to that for 400 nm. Using this property, the concentration of ATP can be quantified as the ratio of GFP fluorescence intensity during excitation at 405 nm to that at 494 nm^11^. On the basis of this previous study, we used a laser to produce excitation at 405 nm and 488 nm and used confocal microscopy to assess the intensity of GFP fluorescence at 513 nm (± 10 nm). We acquired the fluorescent images of each culture before it reached stationary phase^11^ and used these images to calculate the intracellular ATP concentration of each *E. coli* cell in the images relative to a standard curve (Supplementary Fig. 1). We obtained the standard curve by measuring the ratio of ATP concentration and GFP fluorescence intensity upon excitation at 405 nm and 488 nm *in vitro* (see “ATP standard curve” section in “Methods”). Under control concentrations of glucose, the intracellular ATP concentrations of the *E. coli* cells in the culture followed an approximately normal distribution (Fig. 1A). In contrast, under glucose-deficient conditions, most cells in the culture exhibited low intracellular ATP concentrations. However, a small subpopulation retained high ATP concentrations, demonstrated by a long tail in the histogram (Fig. 1B).

We then evaluated the diversity of the overall *E. coli* population by measuring the skewness of the distribution of intracellular ATP concentration. The skewness of the histogram acquired from control conditions was close to 0 (i.e., − 0.273; randomization test, *P* = 1.00000), whereas that under glucose-deficient conditions was significantly larger than 0 (i.e., 0.972; randomization test, *P* = 0.00000). Therefore, intracellular ATP concentrations varied little among the members of *E. coli* populations grown in glucose-sufficient environments, whereas intracellular ATP concentration showed marked diversity among individuals grown under glucose-deficient conditions, as noted previously^11^. These findings validated the conditions (e.g., temperature, glucose concentrations) that we used in all following experiments.

### Creating cellular lineages

**Figure 2:**
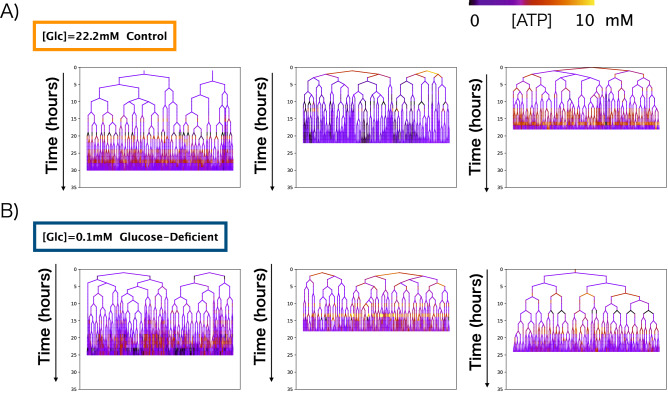
Cell lineages of individual microcolonies under control and glucose-deficient conditions. Cellular lineages acquired by using our custom microfluidic device under (**A**) control conditions (22.2 mM glucose, n = 3) and (**B**) glucose-deficient conditions (0.1 mM glucose, n = 3). The colors indicate the intracellular ATP concentrations of individual cells along a 0- to 10-mM gradient.

To follow chronologic changes in the intracellular ATP concentration of individual cells, we used custom microfluidic devices (Supplementary Fig. 2) to continuously culture *E. coli* populations for 20–30 h in control and glucose-deficient mediums. We then acquired fluorescence images hourly, compartmentalized individual cells by using Schnitzcells^29^, and acquired the intracellular ATP concentration of each *E. coli* cell by using the standard curve (Supplementary Fig. 1). By measuring the changes in ATP concentration of individual members of the population, we constructed cellular lineages for both glucose-deficient conditions (Fig. 2A, Supplementary table 1) and control conditions (Fig. 2B, Supplementary table 1). In general, the constructed cellular lineages showed that as the population became larger, diversity in intracellular ATP concentrations gradually emerged among individuals within that population.

### Cell-to-cell communication is a promising mechanism through which *E. coli* populations might generate ATP concentration diversity

To investigate whether the diversity in intracellular ATP concentration under glucose-deficient conditions is due to stochastic state switching, we constructed Hidden Markov Models (HMMs)^17^ that described ATP concentrations as binary states (high or low). All HMMs were deemed to have two hidden states^28^, given that ATP production through cellular respiration generally can be split into two modes according to the number of ATP molecules generated^4,6,30^.

We then had these HMMs learn the simplified temporal changes in ATP concentrations of the cellular lineages in the control and glucose-deficient conditions by using a global threshold to allocate all concentrations into one of the two classes and performed randomization tests on the learned HMMs acquired under each condition to determine whether the transition probability differed significantly from a random value. Under control conditions, transition probabilities differed significantly from that of a random one (Supplementary Fig. 3; see Supplementary for specific P values for all edges). Under glucose-deficient conditions, most samples showed transition probabilities that were significantly larger than that of a random one (Supplementary Fig. 4; see Supplementary for specific Ps for all edges). When transition probabilities are significantly larger than that of a random one, we can assume significantly increased transition between states; when transition probabilities are significantly smaller, transition between states is significantly reduced. As a result, most samples acquired in this experiment had significant transition probabilities but not condition-specific qualities. Therefore, for each *E. coli* population, factors that contributed to stochasticity existed, but we were unable to confirm the presence of stochastic factors that specifically contributed to diversity in intracellular ATP concentration under glucose-deficient conditions.

Next, we considered whether periodic oscillation contributed to the emergence of diversity in intracellular ATP concentration in *E. coli* populations. To this end, we extracted the principal amplitude and frequency from the chronologic change in ATP concentration of individual *E. coli* cells (Supplementary Fig. 5). Using the aforementioned global threshold for ATP concentration, we grouped individual cells into two classes and compared them. We found that frequency and amplitude differed between the high-ATP and low-ATP classes under control conditions but not under glucose-deficient conditions (Supplementary table 2; Supplementary Fig. 6). Therefore whereas periodic oscillation of intracellular ATP concentration may exist under control conditions, this mechanism is not responsible for creating the diversity in intracellular ATP concentration among single *E. coli* cells under glucose-deficient conditions.

To assess whether the mechanism of cellular age might underlie the emergence of ATP concentration diversity, we first calculated the cellular age of individual cells under control and deprived conditions according to cellular lineage. For *E. coli*, we equated cellular age with the number of times a cellular pole underwent cellular division^20^. We then classified individual cells into the same aforementioned binary classes of high and low ATP phenotypes and evaluated whether cellular age differed between these classes. However, cellular age did not differ significantly between low-ATP and high-ATP classes under either experimental condition (control: *P* = 0.0730 [Mann–Whitney test]; glucose-deficient: *P* = 0.199 [Mann–Whitney test]; Supplementary Fig. 7). Therefore, an individual cell’s age did not contribute to the diversity in intracellular ATP concentration under glucose-deficient conditions.

**Figure 3:**
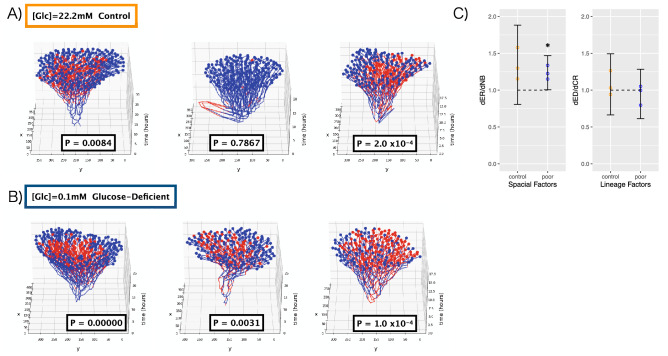
Spatial correlation within lineages grown under glucose-deficient conditions. Three-dimensional cellular lineages of *E. coli* acquired under (**A**) control conditions (22.2 mM glucose) and (**B**) glucose-deficient conditions (0.1 mM glucose) obtained by triplicate experiment. Individual *E. coli* cells were color-coded: red, high intracellular ATP concentration; blue, low intracellular ATP concentration. Individual P values for spatial localization are shown. (**C**) Effects that contribute to spatial localization^28^ of ATP classes. By quantifying the average difference in phenotype among cells closest to a specific individual cell (dNB) and the sister cell of that closest cell (dER), we assessed whether spatial proximity contributed to spatial correlation (dER/dNB). However, for lineage factors, we measured the average difference in phenotype between the closest relative of each individual cell (dCR) and a cell at an equidistant position (dED). The larger the phenotypic difference of the cell at an equidistant position, the stronger the effect of lineage in promoting spatial correlation. The error bar shows the 95% confidence interval in a *t*-distribution. *, *P* < 0.05; H0, dER/dNB = 1 or dED/dCR = 1.

Finally, we tested whether the mechanism of cellular transmission had any role in the emergence of ATP concentration diversity in *E. coli* cells. First, using the same binary classes of high and low intracellular ATP, we tested whether a population showed spatial correlation^28^ (Supplementary Fig. 8) and found statistically significant spatial localization of cells of the same class (Fig. 3A, B). The 3 replicates in this experiment correspond to the 3 plots per condition (Fig. 3A, B). Statistical methods for analyzing the influence of cellular transmission on phenotypic heterogeneity account for the effects of both cell lineage and spatial factors^28^. The method we used is based on the spatial arrangement of individual cells and the cell lineage. The influence of lineage factors is quantified as the ratio of phenotypic differences between the most closely related cells (dCR) and other cells spatially equidistant from them (dED) (i.e., the dED/dCR ratio), whereas the influence of spatial factors is estimated as the ratio of phenotypic differences between the spatially closest cells (dNB) and their spatially more distant sister cells (dER) (i.e., the dER/dNB ratio). When lineage factors have little to no influence and the influence of spatial factors is significantly higher, cellular transmission is presumed to be responsible for the heterogeneity in phenotype. Using this method, we firstly quantified the effect of lineage factors, i.e., dED/dCR, and revealed that lineage factors did not contribute to localization under either experimental condition (Fig. 3C right). In contrast, spatial proximity factors quantified as dER/dNB played a role in creating this spatial localization of ATP classes specifically under glucose-deficient conditions (Fig. 3C left). Although in many flat cultures of *E. coli* the sister cells are adjacent to each other, this method utilizes additional cells, such as the second-generation daughter cells coming from a single mother cell, allowing for more variability in spatial proximity. Spatial localization of phenotypes specifically caused by spatial proximity factors in populations under glucose-deprived conditions show that mechanisms that depend on the exchange of molecules such as cellular transmission are responsible. These results imply that mechanisms that depend on the exchange of molecules—that is, cellular transmission—underlie the diversity in intracellular ATP concentrations among *E. coli* cells in a glucose-deprived environment.

### Intercellular diversity in intracellular ATP concentration can be reproduced in glucose-corrected supernatants

According to the results in the previous sections, cellular transmission accounts for the emergence of diversity in intracellular ATP concentration among *E. coli* cells. This diversity is due to the observed spatial localization created through spatial proximity that was specific to the glucose-deficient condition; therefore, our next step was to experimentally validate cellular transmission as the key factor in the emergence of ATP diversity in *E. coli*.

**Figure 4:**
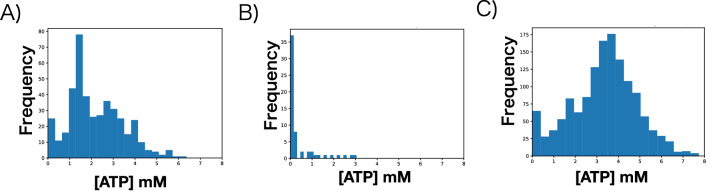
Comparison of intracellular ATP in cells cultured in glucose-corrected supernatants, ATP-supplemented control medium, and glycerol-supplemented control medium. (**A**) Distribution of intracellular ATP concentration in individual cells grown in a medium based on supernatants of glucose-deficient conditions (skewness = 0.559, *P* = 0.00001, n = 425). (**B**) Distribution of intracellular ATP concentration in individual cells grown in glucose control medium supplemented with 10 mM ATP (skewness = 2.070, *P* = 0.00000, n = 38). (**C**) Distribution of intracellular ATP concentration in individual cells grown in glucose control medium supplemented with 10 mM glycerol (skewness = − 0.1472, *P* = 0.98711, n = 1383).

Considering metabolic diversity to be due to cellular transmission, we thought it probable that the molecules involved might be released into the extracellular space. To this end, we collected supernatants from *E. coli* cultures grown under glucose-deficient conditions, added glucose to these supernatants until the control glucose concentration was achieved, and cultured new *E. coli* populations in the glucose-corrected mediums (Fig. 4A). When we measured the intracellular ATP concentration of individual cells before they reached the stationary phase, the acquired *E. coli* population had a long-tail distribution similar to that of the glucose-deficient conditions (Fig. 4A) and a skewness that was significantly larger than 0 (skewness, 0.559; *P* = 0.00001), consistent with the measured diversity. These findings indicate that cellular transmission molecules released into the extracellular space can indeed cause diversity in the intracellular ATP concentration of *E. coli* cells regardless of the amount of glucose in their environment.

### The availability of extracellular ATP can lead to extreme diversity in intracellular ATP levels

Given that *E. coli* populations under glucose-deficient conditions release into the proximal extracellular space cellular transmission molecules that affect the intracellular ATP concentrations of individual cells, we used gas chromatography–mass spectroscopy (GC–MS) techniques to measure all metabolic components (Supplementary Data 1) of the supernatants from cultures grown under glucose-deficient conditions (Supplementary Fig. 9). Glycerol and metabolites containing phosphate were the only components whose molecular concentrations under glucose-deficient conditions were as high as those under control conditions. However the $\hbox {OD}_{600}$ of the cultures grown under control was twice that under glucose-deficient conditions, thus suggesting that each individual cell releases many more metabolites under glucose-deficient conditions. From GC-MS analysis alone, the specific metabolites involved in this phenomenon were unclear, especially those containing phosphate. We therefore focused on the metabolite ATP, which contains phosphate and functions through various mechanisms to activate and repress its own production^31^. The ATP concentration was 57.19 ± 6.88 nM in supernatants from cultures grown under control conditions and 55.00 ± 14.7 nM in those from glucose-deficient conditions. These findings support the notion that indeed *E. coli* cells release ATP into their extracellular environment under both culture conditions. We then normalized the ATP concentration of the supernatant according to the $\hbox {OD}_{600}$ of the sample, yielding a value of 164.1 ± 22.6 nM $\hbox {OD}_{600}^{-1}$ under control conditions and of 5911 ± 2222 nM $\hbox {OD}_{600}^{-1}$ under glucose-deficient conditions. These results indicate that *E. coli* cells release more ATP under glucose-deficient conditions.

Next, we sought to recreate the emergence of intracellular ATP diversity through the addition of extracellular ATP (eATP). Adding 10 mM ATP to the control medium of *E. coli* cultures yielded a distribution of intracellular ATP concentrations that was similar to that of glucose-deficient conditions, where only a few *E. coli* cells retained high intracellular ATP concentrations (Fig. 4B); the skewness of this distribution was significantly larger than 0 (skewness, 2.070; *P* = 0.00000). In contrast, adding 10 mM glycerol, another substance highly released by *E. coli* cells grown under glucose-deficient conditions, to control conditions failed to achieve a long-tailed distribution in intracellular ATP concentration (Fig. 4C), and the resulting skewness was smaller than 0 (skewness, − 0.1472, *P* = 0.98711 > 0.05). Therefore we revealed that diversity in intracellular ATP concentration among *E. coli* cells in glucose-deficient environments arises through the mechanism of cellular transmission due to the extracellular release of ATP by a specific cellular subpopulation.

## Discussion

This study focused on quantifying and analyzing how diversity in intracellular ATP concentration emerges in *E. coli* under glucose-deficient conditions, under which most individual cells have low ATP concentrations yet a subpopulation retains high levels of intracellular ATP. We here revealed that the emergence of diversity was due to the release of ATP into the extracellular environment through the mechanism of cellular transmission. Despite many reports on the stochasticity of metabolic activities^5,15,32,33^, we were unable to confirm stochasticity in relation to a specific culture condition for *E. coli*. Likewise, our findings did not support periodic oscillation as being the mechanism specific to diversity under glucose-deficient conditions. Periodic oscillation of other metabolites in *E. coli* has been reported^19^.

In contrast, from the perspective of the general physiology of *E. coli*, the diversity of intracellular ATP concentration under glucose-deficient conditions may also be considered to be caused by differences in growth rate or growth state (growth vs. stationary phase) at the start of the experiment. To determine whether these factors had an effect, we first measured the growth rate of *E. coli* under control and glucose-deficient conditions and found no significant differences between them (Supplementary Fig. 10, Mann–Whitney U test, *P* = 0.33126 > 0.05). Next, the effects of the different physiological states of *E. coli* can be considered based on the setup of the incubation time. Here, *E. coli* was cultured overnight for 16 h and 3-h preincubation prior to assessing the cell lineage, thus revealing the temporal change in ATP concentration, which is considered to have reached an exponential growth phase by the start of the cell lineage measurement, regardless of control or glucose-deficient conditions. We conclude that the diversity in ATP concentration under glucose-deficient conditions is not due to simple factors, such as differences in the growth rate or physiological state of *E. coli*.

Mechanisms that are heavily dependent on the initial state of the bacterium, such as cellular age and periodic oscillation, might be expected to induce an initial difference. However, our results suggest that differences in initial culture conditions do not affect ATP concentration heterogeneity. These data suggest that although initial differences in cellular state may exist at the time of analysis, they are not responsible for causing heterogeneity in intracellular ATP concentrations.

For cellular transmission to maintain high ATP concentrations in nearby cells only, the transmission molecules themselves must remain in close proximity to the releasing cell, without diffusion throughout the entire population. According to recent reports, the transmission of metabolites similar to ATP is localized and affects only cells in the immediate vicinity of those releasing the transmitted molecule^34^. Our results suggest that a metabolite such as ATP acts as a cellular transmission molecule in *E. coli*, and we thus infer the possibility of a mechanism of diversity that utilizes ATP as a localized transmission molecule between cells and thus promotes differences of intracellular ATP concentration between individual cells that are prone to receiving such molecules.

We now consider the biochemical mechanisms through which an ATP cellular transmission mechanism causes only specific cells to maintain high concentrations of intracellular ATP. In general, intracellular ATP is produced through glycolysis, which produces intermediate metabolites to fuel the tricarboxylic acid (TCA) cycle. The ATP thus produced is utilized in many biochemical reactions throughout the cell, such that ATP can influence many biologic phenomena. Given that metabolic processes themselves are among these ATP-mediated biochemical reactions, ATP is not the only product of cellular respiration. For example, as intracellular ATP decreases, various intermediate metabolites are activated^35^, and the expression levels of enzymes mediating glycolysis and the TCA cycle increase^31^. These situations support the possibility that ATP regulates its own production. In addition, notwithstanding the presence of intracellular ATP, *E. coli* cells are thought to actually utilize eATP. For example, eATP levels increase during the growth phase, but as carbon sources decrease in the medium, eATP likewise decreases^30,36^, thus demonstrating the context of eATP usage under carbon source deprivation. Given our current findings, we surmise a similar relationship between extracellular release and intake of ATP in *E. coli*.

Adding 10 mM ATP to the control medium yielded other interesting findings. *E. coli* cells cultured in this medium showed similarly high skewness for distribution of the intracellular ATP concentration, but the fraction of cells that retained high ATP concentrations was smaller compared with that in other conditions (Fig. 4B). In addition, even when eATP was abundant, most cells had low intracellular ATP concentrations. However, since a number of *E. coli* cells were visible, they were not dying. Therefore, our results suggest that, regardless of glucose available in the extracellular space, when eATP is abundant, *E. coli* can utilize it yet retain low ATP concentrations intracellularly.

In light of our results regarding spatial correlation generated through the spatial proximity of cells under glucose-deficient conditions, we suggest a mechanism whereby the majority of the population diverts from glucose consumption to utilizing eATP released from other cells, thus dramatically repressing the respiratory system. However, under this compensatory mechanism, a subpopulation of cells continues to intake glucose and thus retains high intracellular ATP concentrations, consequently yielding diversity in intracellular ATP concentration throughout the entire population.

Then, why is the diversity in levels of intracellular ATP necessary for *E. coli*? Given the necessary role of ATP in inducing many biochemical reactions, it is logical to think that having an abundance of intracellular ATP is beneficial to individual cells. However, glucose-deficient conditions likely provide insufficient resources for producing ATP, and it would be difficult to distribute the available environmental glucose to all individual cells in the population for each to produce ATP independently. If all the glucose available in the environment were distributed equally among all individual cells in the population, extinction of the entire population can be expected when the population is in an environment that lacks the bare minimum of carbon sources necessary for each individual to sustain life. We predict that to avoid such scenarios, *E. coli* harbors a mechanism through which most cells convert to consuming eATP, but a subpopulation of cells continues to produce ATP from the glucose available, as though under glucose-replete conditions. Under this strategy, the majority of the population feeds on the metabolic intermediates released by the cells in the glucose-using subpopulation. This scenario can be considered as a metabolic division of labor that keeps the overall population from becoming extinct.

We have shown that the strategy that *E. coli* populations use to keep some individual cells alive during glucose deficiency involves ATP-mediated intercellular communication. However, a quantitative understanding of the underlying mechanism requires clarification of the spatial distribution of the eATP concentration, its stability, the threshold glucose concentration necessary for diversity generation, the time constants of the reaction, and the amount of products generated under the reaction kinetics inside the cell. In addition, the effects of various extracellular environmental factors—including pH, oxygen concentration, and cell density, as shown in this study—on the exchange of ATP outside the cell should also be considered. Findings from these quantitative perspectives likely will improve our understanding of survival strategies in microorganisms.

To summarize, our current findings suggest that for environments where glucose resources are limited, clonal populations of *E. coli* employ metabolic division of labor, such that individual cells solely consume either eATP or glucose. This specialization results in the diversification of intracellular ATP concentration within the population. We consider that this mechanism is adopted as a tactic to protect against extinction of the entire *E. coli* population and maintain population growth.

## Methods

### Bacterial strains, plasmids, and media

Unless stated otherwise, all experiments used *E. coli* MG1655 strain (a gift from Professor Yuichi Wakamoto at Tokyo University) as wild-type (WT). We used ligation-independent cloning PCR^37^ to insert the QUEEN-2m construct into the ptqk5 plasmid vector, a low-copy modified version of the original pRSetB–QUEEN-2m plasmid^11^ obtained from the *Bacillus* PS3 strain and encoding a truncated protein $\epsilon$ of FoF1 ATPase, to generate the resulting ptqk5–QUEEN-2m plasmid. QUEEN-2m protein purification was performed by using the *E. coli* JM109 strain.

M9 medium (1x M9 salts, 2 mM MgSO4, 0.1 mM CaCl2; Sigma-Aldrich) supplemented with 1% thiamine and 0.1 mM glucose was used for glucose-deprived conditions. M9 medium supplemented with 1% thiamine and 22.2 mM glucose was used for the glucose control condition. Luria broth medium (LB medium; Merck) was used for protein purification protocols. For experiments assessing specific agents, such as 10 mM ATP (TOYO B-Net) and 10 mM glycerol (Wako), the substance of interest was combined with the various components of the glucose control medium such that the described concentrations were maintained. All mediums included ampicillin as an antibacterial agent.

### Microscopy

Bulk-culture snapshot and time-lapse imaging were performed by using a fully automated inverted microscope (model IX81, Olympus) with a 100$\times$ oil objective lens (Olympus), confocal scanning unit (model CSU-X1, Yokogawa), and a digital complementary metal-oxide semiconductor (CMOS) camera (ORCA-Flash4.0, Hamamatsu). Time-lapse images were acquired hourly for 20–40 h (Supplementary Fig. 11). Fluorescent imaging used 488LP, 509/22 dichroic mirror cubes (Olympus) and 488- and 405-nM lasers mounted onto the inverted microscope (model IX81, Olympus). The laser intensity was uniformly 50 mW, and the exposure time was 1000 ms for excitation with the 405-nm laser and 200 ms for excitation with the 488 nm laser. Focus was maintained manually by using live bright-field images. The ${\upmu }$-manager program was used to control the microscope^38^.

For bulk snapshots of single-lineage cultures, all images were taken under $30\,\,^{\circ }\hbox {C}$ conditions. Cultures were grown in the appropriate glucose-treated M9-based medium from single colonies on LB agar plates streaked with transformed samples or with glycerol stocks. 3 mL of cultures were grown overnight for 16 h, during which *E. coli* cultures typically reach stationary phase. Then, cultures were diluted to $\hbox {OD}_{600}$ 0.05 and cultured for 3 h with shaking at 220 RPM until the culture OD600 was 0.2, when *E. coli* cultures usually are in the exponential growth phase. Then the *E. coli* cells in 5 μL of culture were immobilized on cover glasses by using thin pads of agarose; treated cover glasses were left to dry for about 30 minutes before imaging.

### Construction of the microfluidic device and its use in microscopy

For long-term microscopy, we used custom-designed microfluidic devices, as shown in Supplementary Fig. 2. The dimensions of the agarose pad channel was approximately 12 mm $\times$ 4 mm $\times$ 0.28 mm, which has an additional channel for medium, 8 mm $\times$ 1 mm $\times$ 0.28 mm, positioned on top. A circular channel for negative pressure surrounds the above channels with a dimension of 20 mm $\times$ 0.07 mm. Molds for the devices were created from adhesive polyethylene terephthalate that was cut into appropriate sizes by using a cutting plotter (Graphtec). The molds were then filled with polydimethylsiloxane (PDMS; Dow Corning Toray), which was hardened at $60^{\circ }\hbox {C}$ overnight. The devices were cut from the molds by using a razor blade, and a thin PDMS film was attached to device inlets and outlets by using standard ion-bombardment techniques and an ion bombarder (model PIB-10, Vacuum Device Incorporated). After adhesion of the film, a biopsy punch was used to create ports for channels to apply medium and negative pressure. Time-lapse microcolony images of continuously growing *E. coli* cultures were obtained under $30^{\circ }\hbox {C}$ incubation conditions by using a stage-top incubator (Tokai hit) and the microfluidic device; Z-axis drift was corrected manually as needed.

### ATP standard curve

*E. coli* JM109(DE3) cells were transformed with the pRSetB–QUEEN-2m vector^11^ and cultured overnight at $37^{\circ }\hbox {C}$; 1 mM isopropyl $\beta$-D1-thiogalactopyranoside (Wako) was added at 3 h prior to harvest. Protein purification of QUEEN-2m from harvested cells was performed as described previously^39^, and an ATP standard curve was drawn according to previously described methods^11^.

### GC-MS

Extracts were obtained from the supernatants of 3 mL of overnight cultures of *E. coli* in M9 medium containing 0.1 or 22.2 mM glucose. All samples were centrifuged at 10,000 $\times \mathrm{{g}}$ for 5 minutes to create bacterial pellets; supernatants were passed over $0.22-{\upmu }\hbox {m}$ filters (Millipore, Merck) to remove bacterial debris and then diluted 90-fold. For each sample, $100\,{\upmu }\hbox {L}$ of supernatant was concentrated, derivatized by using N-Methyl-N-(trimethylsilyl)trifluoroacetamide (MSFTA) and methylhydroxylamine hydrochloride, and analyzed via GC–MS (GCMS-TQ8030, Shimadzu) using 2-isopropylmalic acid as the internal standard. All metabolites measured were shown in Supplementary Data 1.

### ATP assay

ATP assays (ATP Assay Kit, Sigma-Aldrich) used the extracts from 3 mL of overnight cultures of *E. coli* grown in M9 medium supplemented with 0.1 or 22.2 mM. For low-$\hbox {OD}_{600}$ samples (i.e., *E. coli* grown in 0.1 mM glucose), samples were centrifuged once at 10,000 $\times \mathrm{{g}}$ for 5 minutes and then diluted with medium to create a 6-fold concentrated sample. The $\hbox {OD}_{600}$ of each overnight culture and the ATP concentrations of its supernatant samples were measured to calculate the nM $\hbox {OD}_{600}^{-1}$ of each biological replicate under both conditions.

## Statistics and reproducibility

### Skewness calculations

The skewness of a histogram was calculated according to the Fisher–Pearson coefficient of skewness as in the equation below by using the pandas package in Python^40^.

1 $$\begin{aligned}&g_{1}=\frac{m_{3}}{m_{2}^{3 / 2}} \end{aligned}$$

where,

2 $$\begin{aligned}&m_{i}=\frac{1}{N} \sum _{n=1}^{N}(x[n]-\bar{x})^{i} \end{aligned}$$

### Determination of skewness significance

For all histograms, skewness significance was analyzed by using randomization tests. The skewness of 100,000 normal distributions was sampled by using the mean, standard deviation, and sample size ($\mu ,\sigma , n$) of the original histogram. We then compared the skewness of the sampled distributions with that of the original histogram to quantify the rarity of skewness of the original histogram.

### Selection and analysis of microcolonies

For analysis, we selected colonies for which all progeny stayed in the field of view and for which fluorescence data were viable for segmentation by using Schnitzcells^29^. When fluorescence images did not support segmentation, we used images from previous time points as place holders to maintain lineage tree structure; data from place-holder images were excluded from analysis.

### Analyses regarding the four theories of heterogeneity

All fluorescence images were segmented and tracked using Schnitzcells^29^. Lineage data were reconstructed in Python by using a custom code. Except for the analysis regarding spatial correlation, all analyses were conducted by using R or custom Python codes. Spatial correlation analyses were conducted by using a MATLAB code^28^ that measured spatial correlations and affecting factors within lineage trees.

ATP concentration data obtained from the lineage trees for the glucose-deficient condition were separated into two phenotypic classes relative to the threshold value that split the total ATP distribution among cells under glucose deprivation into two mixed Gaussian distribution with the total maximum likelihood.

### Construction of HMMs

The basic methods and ideas for these studies were derived from previous research^17^. The HMM library ’aphid’ in R was used to construct HMMs^41^. Two-state HMMs were used given the assumption that ATP production is dependent on the respiratory system involved. The output state of the model was either high ATP or low ATP. Learned state transfer vectors were created by applying the Baum–Welch algorithm (Supplementary Fig. 12) to the experimental data from individual lineages, which were split according to a threshold value into the two output states.

For all models, the statistical significance of the probability of transition between the two states was tested by using randomization tests. These tests compared 4000 different HMMs, in which the learning state transition vectors for each model were randomly shuffled. When a transition probability obtained from the experimental data was smaller than the 5th percentile of the randomized distribution, that transition probability was considered significantly small. Similarly, when the transition probability obtained from the experiment was larger than the 95th percentile of the randomized distribution, that transition probability was considered significantly large.

### Gaussian process regression for changes in ATP concentration

The Gaussian process regression library ’GPFit’ in R was used to construct HMMs^42^ for these studies; regressions excluded the aforementioned place-holder data. Regression convergence was estimated according to the correlation hyperparameter estimate $\beta$. $\beta _k$ was obtained by using the following equation.

3 $$\begin{aligned} \beta _k = \log _{10}(\theta _k), \end{aligned}$$

where $\theta$ is the hyperparameter set at the *k*-th iteration. The GPFit package utilizes $\beta$ and $\theta$ to determine the negative profile log-likelihood of the current regression and the data. After a maximum of *k* = 10,000 iterations or convergence, the value of $\beta$ is achieved. When the regression did not converge ($\beta$ > 4), that lineage was removed from analysis.

### Analysis of cellular age

*E. coli* cellular age was determined by using the lineage structure and spatial relationships. Specifically, centroids between progenies that shared common grandmothers were used to determine newborn cells, older cells, and the oldest cell. Cellular age was determined as described by Stewart et al.^20^

### Calculation of growth rate

We used the generalized Euler–Lotka equation^43^ to determine the growth rate of *E. coli* populations under control and glucose-depleted conditions. This equation enables the calculation of growth rate of heterogenous populations. It relies on the number of cell divisions ($\Delta$) in each independent lineage rather than on the time frame to calculate the population growth rate, thus allowing it to be robust against incomplete cellular lineages. The generalized Euler–Lotka equation is given as

4 $$\begin{aligned} \lim _{\Delta \rightarrow \infty } \frac{1}{\Delta } \ln \left\langle e^{-\Lambda \sum _{i=1}^{\Delta } \tau _{i}}\right\rangle _{\left\{ \tau _{i}\right\} }=-\ln 2 \end{aligned}$$

where $\Lambda$ denotes the growth rate and $\langle \cdots \rangle _{\{\tau _{i}\}}$ denotes an average over sequences for $\{\tau _{i}\}=\tau _1,\tau _2,\ldots ,\tau _{\Delta }$, the cell division times for each independent lineage. Using this equation, we calculated $\Lambda$ for the change over time in the number of *E. coli* under control and glucose-depleted conditions.

## Supplementary Information

Supplementary Information 1.

Supplementary Information 2.

## Data Availability

The datasets generated and/or analysed during the current study are available in the github repository, https://github.com/funalab/pyCellLineage.

## Code availability

The custom analysis codes created in this study are freely available through github (https://github.com/funalab/pyCellLineage).
