# Supplementary material for: Intercellular interaction mechanisms promote diversity in intracellular ATP concentration in Escherichia coli populations
Source: Sci Rep. 2022 Oct 26;12:17946. doi: 10.1038/s41598-022-22189-x (PMC9605964; doi:10.1038/s41598-022-22189-x)
Supplement: Supplementary file 1 — Supplementary Information 1. [file 41598_2022_22189_MOESM1_ESM.pdf]

Supplementary Figures and Tables : Intercellular Interaction Mechanisms Promote Diversity in Intracellular ATP Concentration in *Escherichia coli* Populations

Ryo J. Nakatani[1], Masahiro Itabashi[1], Takahiro G. Yamada[1,2], Noriko F. Hiroi[3,*], and Akira Funahashi[1,2,*]

[1]Center for Biosciences and Informatics, Graduate School of Fundamental Science and Technology, Keio University, Yokohama, Kanagawa, 223-8522, Japan
[2]Department of Biosciences and Informatics, Keio University, Yokohama, Kanagawa, 223-8522, Japan
[3]Graduate School of Media and Governance, Keio University, Fujisawa, Kanagawa, 252-0882, Japan
[*]*hiroi@bio.keio.ac.jp* and *funa@bio.keio.ac.jp*

Supplementary Figures

1 Calibration curve produced under experimental conditions . . 3
2 Setup and schematics of the microfluidic device used in the experiments. . . . 4
3 Learned Hidden Markov Models (HMMs) and randomization test results for control conditions . . . 5
4 Learned HMMs and randomization test results for glucose-deficient condition . . . 6
5 Individual lineages of all samples . . . 7
6 Comparison of maximum frequency and amplitude of ATP classes under both experimental conditions . . . 8
7 Cellular age distribution among ATP classes under each experimental condition . . . 9
8 Results of randomization tests for spatial correlation of *E. coli* samples grown under control and glucose-deficient conditions . 10
9 Metabolites detected by GC–MS . . . 11
10 Growth rates under control and glucose-deficient conditions . . 12
11 Representative images used for data analysis . . . 13
12 State-transition lineages . . . 14

Supplementary Tables

1 Various quantitative information in each *E. coli* lineages in Fig. 2 of the main body. . . . 15
2 P values of Mann–Whitney test comparing maximum amplitude and frequency between ATP classes under control and glucose-deficient conditions. . . . 16

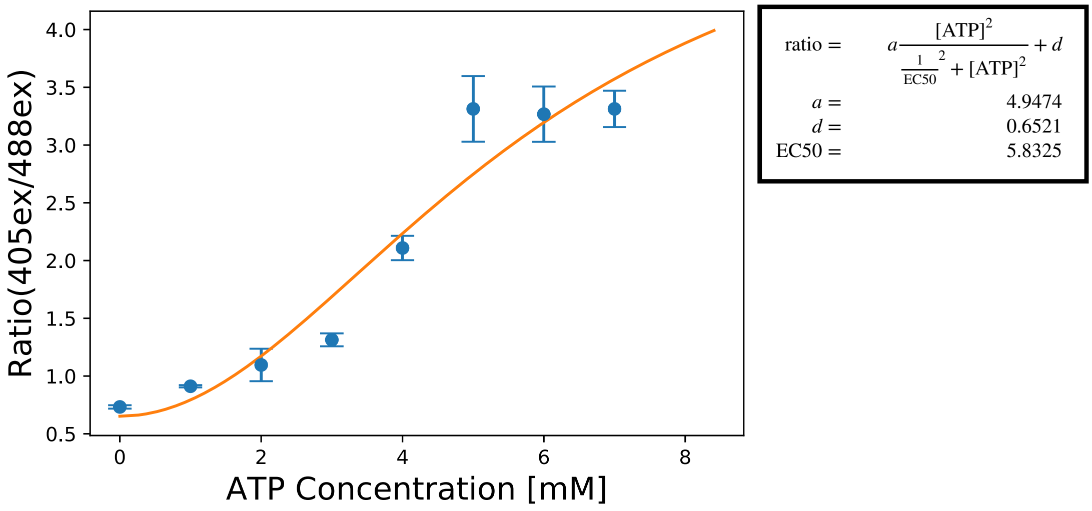

Supplementary Figure 1: Calibration curve produced under experimental conditions

The relationship between ATP concentration and excitation ratio (405 nm/488 nm) in *E.coli* cells bearing a QUEEN-2m construct in a 30 °C environment obtained under our experimental setup. The curve shown is a Hill curve (n = 2) fitted to the data.

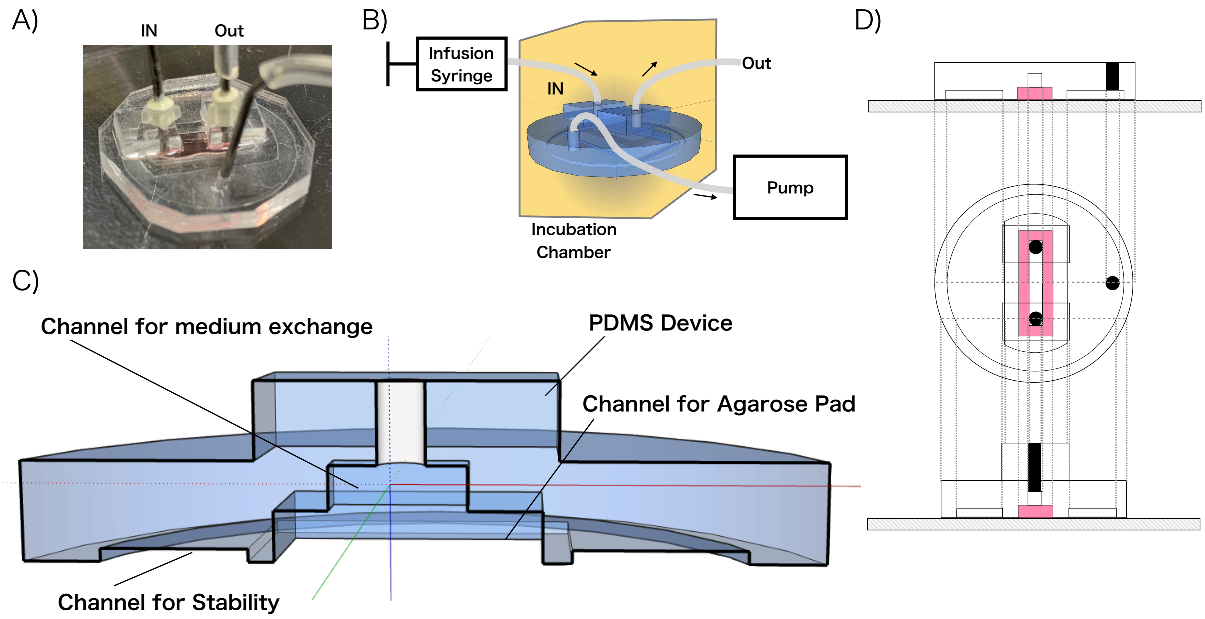

Supplementary Figure 2: Setup and schematics of the microfluidic device used in the experiments.
A) Device setup for observation. The red fluid shows where channels for medium exist. B) The device setup for observation of *E. coli* cells. There are two channels for medium exchange: one for infusion from a syringe, and the other for collection of waste. To stabilize the device, negative pressure is applied to the channel on the edge of the device by using an air pump. A circular channel for negative pressure with a dimension of 20 mm $\times$ 0.07 mm. During culture experiments, the device was set up within a stage-top incubator. C) The device in cross section, showing the role of each channel within the figure. The dimensions of the agarose pad channel was approximately 12 mm $\times$ 4 mm $\times$ 0.28 mm, which has an additional channel for medium, 8 mm $\times$ 1 mm $\times$ 0.28 mm, positioned on top. D) The device cross section compared with the device schematics. The red color indicates the channels, and black spots represent inlet and outlet ports.

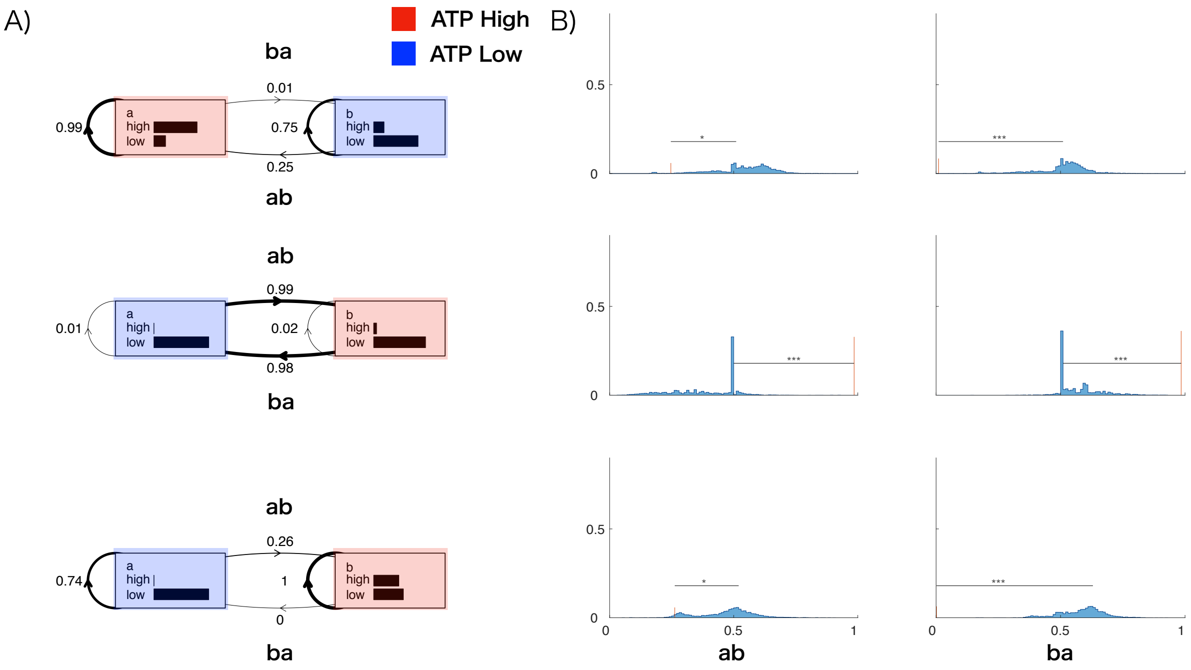

Supplementary Figure 3: Learned Hidden Markov Models (HMMs) and randomization test results for control conditions

Learned two-state HMMs for samples under control conditions (22.2 mM glucose, n = 3). A) A model of learned HMMs based on the Baum–Welch algorithm. Each hidden state is color coded according to its predominant output (blue, low ATP; red, high ATP). B) Results of the transition probability randomization test for each transition probability in the model. Some models exhibit transition probabilities that are significantly smaller than that of a randomly learned model. *, $P < 0.05$; **, $P < 0.01$; ***, $P < 0.001$; H_0, no difference between HMMs learning the experimentally observed transition vector and HMMs learning a randomly reordered version of the experimentally observed transition vector.

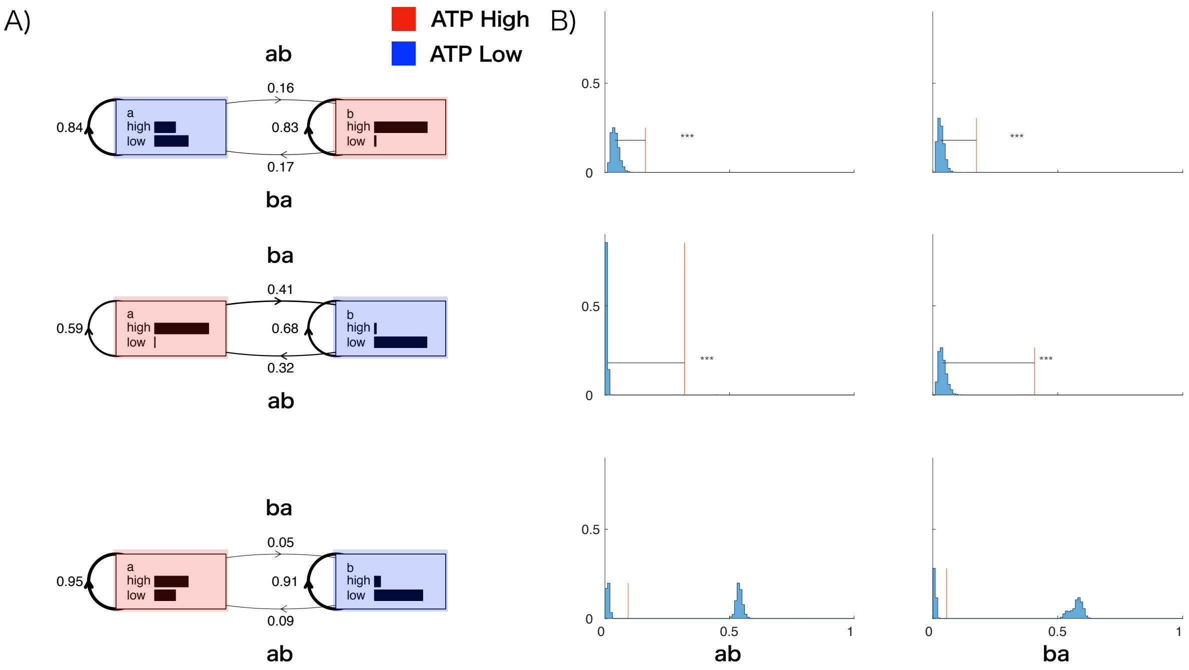

Supplementary Figure 4: Learned HMMs and randomization test results for glucose-deficient condition

Learned two-state HMMs for each sample obtained under glucose-deficient conditions (0.1 mM glucose, n = 3). A) A model of learned HMMs based on the Baum–Welch algorithm. Each hidden state is color-coded according to its predominant output (blue, low ATP; red, high ATP). B) Result of the transition probability randomization test for each transition probability in the model. For all models, the experimentally observed transition probability ab, ba is not significantly different from a randomly learned probability. *, $P < 0.05$; **, $P < 0.01$; ***, $P < 0.001$; H_0, no difference between HMMs learning the experimentally observed transition vector and HMMs learning a randomly reordered version of the experimentally observed transition vector.

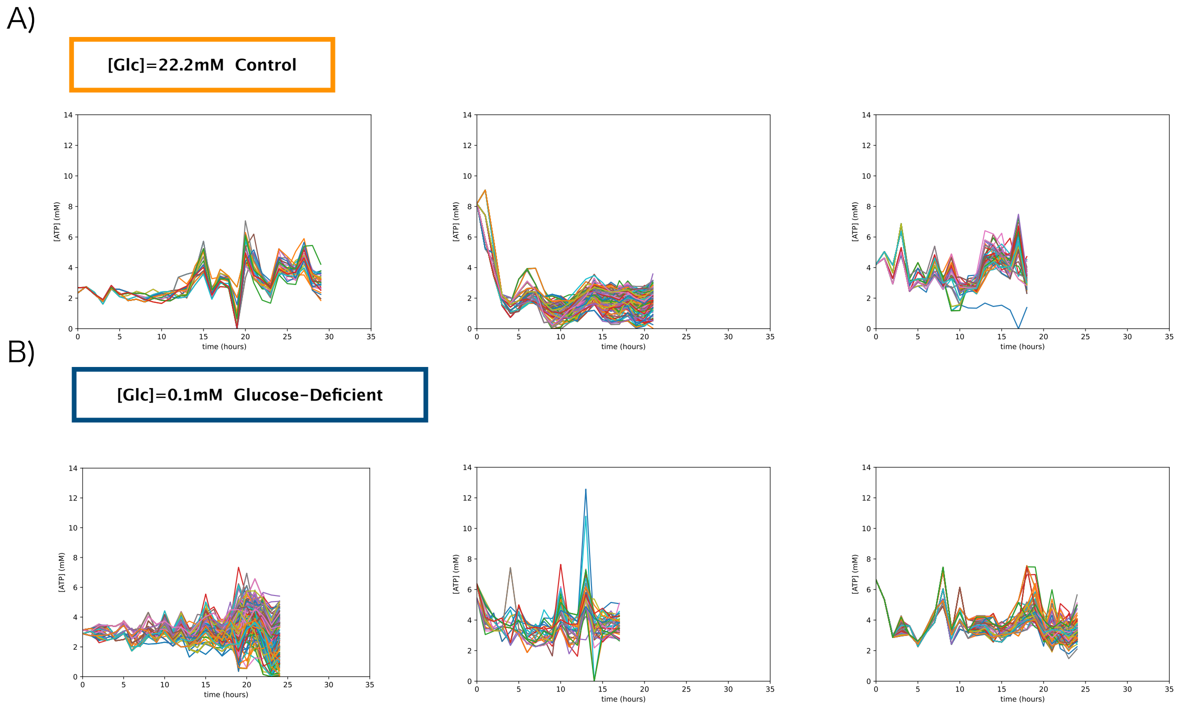

Supplementary Figure 5: Individual lineages of all samples
Results of Gaussian process regression for chronologic changes in intracellular ATP concentration under A) control (22.2 mM glucose) and B) glucose-deficient (0.1 mM) conditions (n = 3 for each). Within each panel, individual lineages are color-coded.

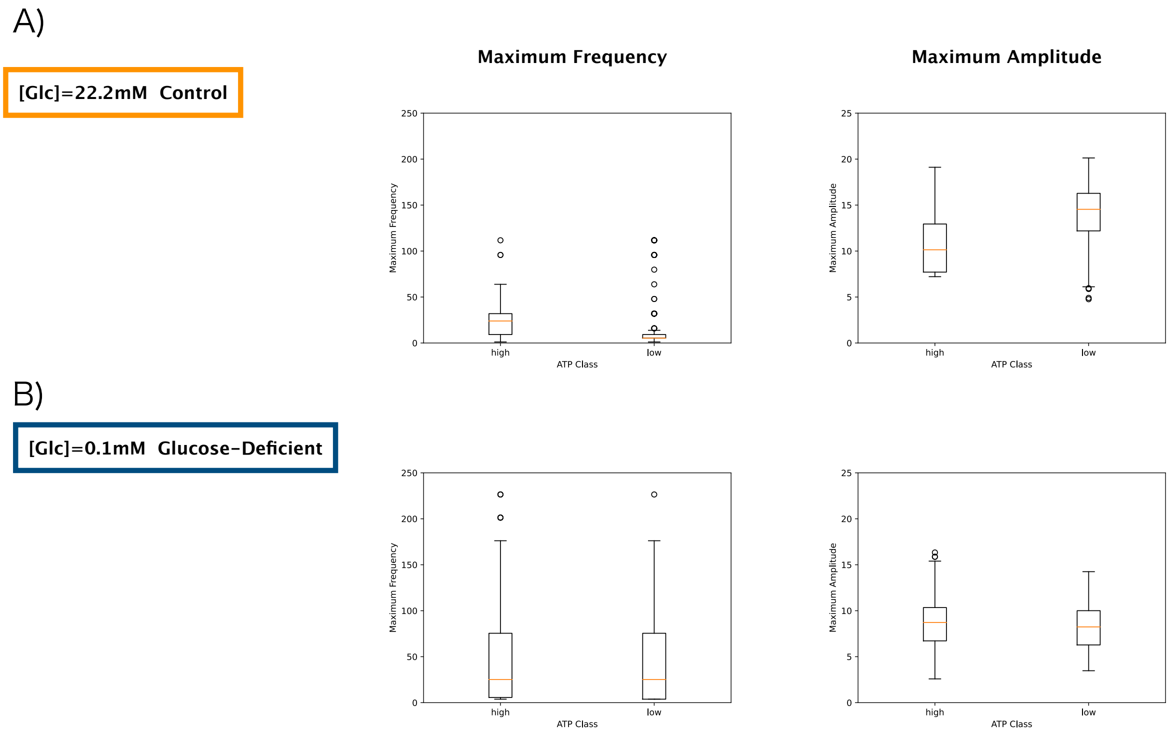

Supplementary Figure 6: Comparison of maximum frequency and amplitude of ATP classes under both experimental conditions

The amplitudes and frequencies plotted for each ATP class under control and glucose-deficient conditions. A) Frequencies (left) and amplitudes (right) plotted for each ATP class for samples obtained under control conditions (22.2 mM glucose, n = 3). B) Frequencies (left) and amplitudes (right) plotted for each ATP class for samples obtained under glucose-deficient conditions (0.1 mM glucose, n = 3). Classes were compared by using the Mann–Whitney test; P values are reported in Supplementary table 2.

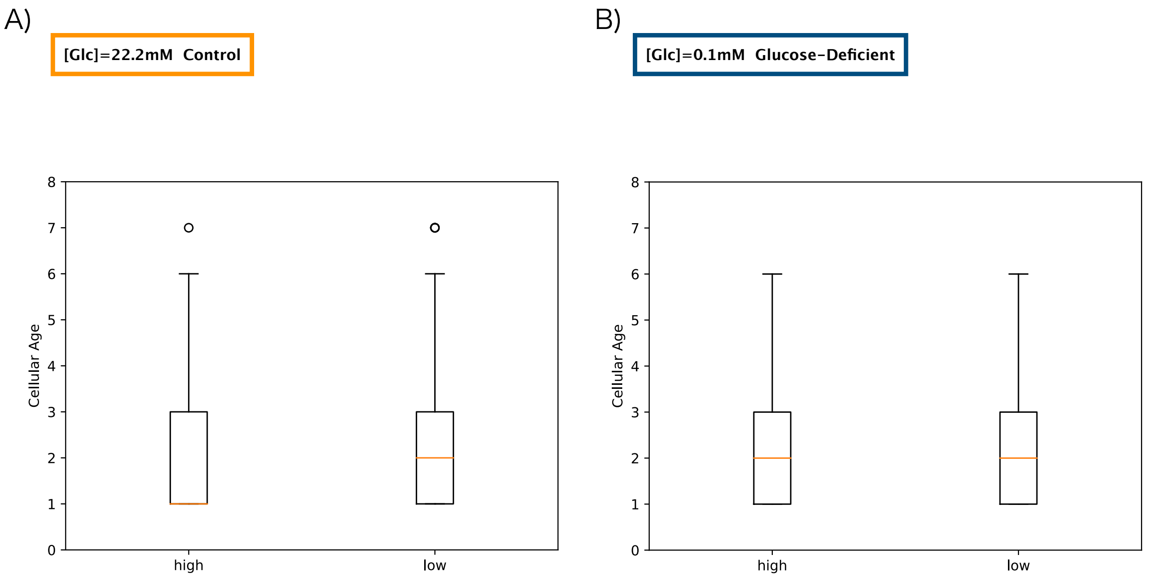

Supplementary Figure 7: Cellular age distribution among ATP classes under each experimental condition
Plots of cellular age according to ATP class for samples under control and glucose-deficient conditions. A) Cellular age according to ATP class for samples obtained under control conditions ($n = 3$); $P = 0.0730$ (Mann–Whitney test). B) Cellular age according to ATP class for samples obtained under glucose-deficient conditions ($n = 3$); $P = 0.199$ (Mann–Whitney test).

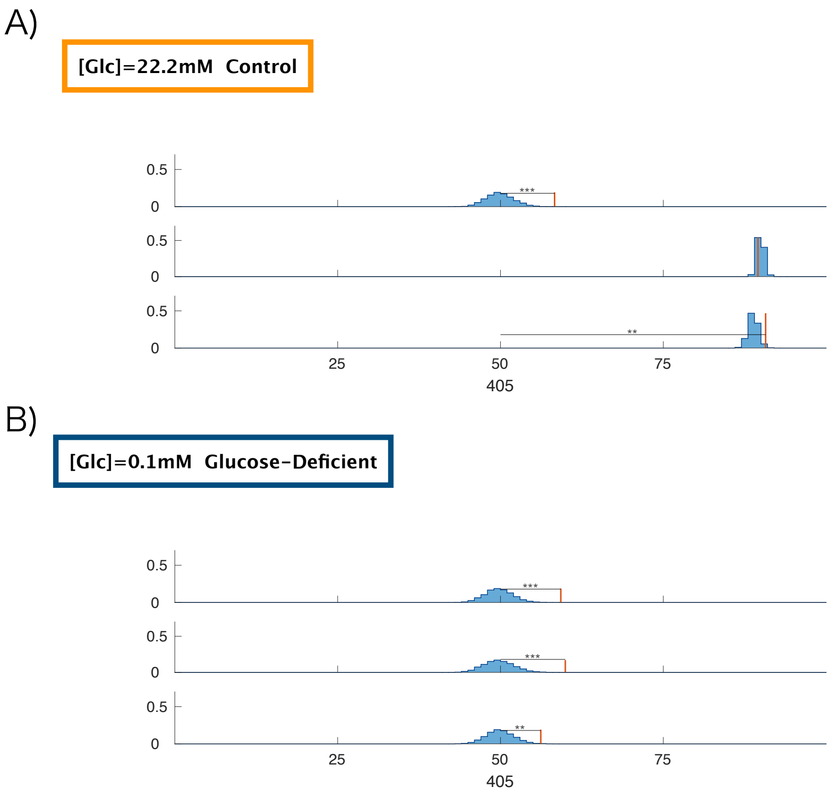

Supplementary Figure 8: Results of randomization tests for spatial correlation of *E. coli* samples grown under control and glucose-deficient conditions A) Distribution under the control condition (n = 3) when calculating the percentage of neighbors of the same phenotype for phenotypes randomly interchanged among individual cells. The experimentally obtained value is indicated in the figure. B) Distribution under the glucose-deficient condition (n = 3), when calculating the percentage of neighbors of the same phenotype for phenotypes randomly interchanged among individual cells. The experimentally obtained value is indicated in the figure. Distributions reflect 10,000 repetitions, with individual phenotypes randomly interchanged within a given sample. The orange bar shows the experimentally observed percentage of neighbors of the same phenotype. *, $P < 0.05$; **, $P < 0.01$; ***, $P < 0.001$; H_0, the experimentally observed percentage of neighbors of the same phenotype is similar to that of a random one.

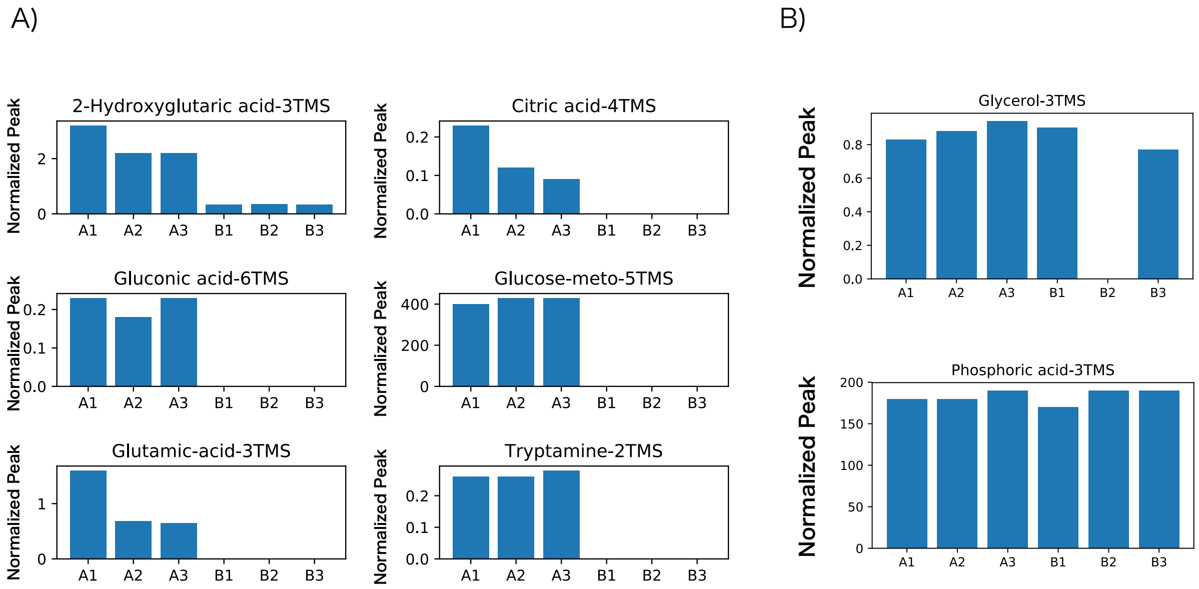

Supplementary Figure 9: Metabolites detected by GC–MS

Metabolites detected in the supernatants of *E. coli* populations cultured in control or glucose-deficient media (Supplementary Data 1, i.e., no metabolites other than these were detected in any of the samples). A) Metabolites detected only under control conditions. B) Metabolites detected under control and glucose-deficient conditions. In each graph, A1, A2, and A3 are levels of the target metabolite in supernatants of cultures under control conditions (22.2 mM glucose), and B1, B2, and B3 are levels in supernatants of cultures under glucose-deficient conditions (0.1 mM glucose).

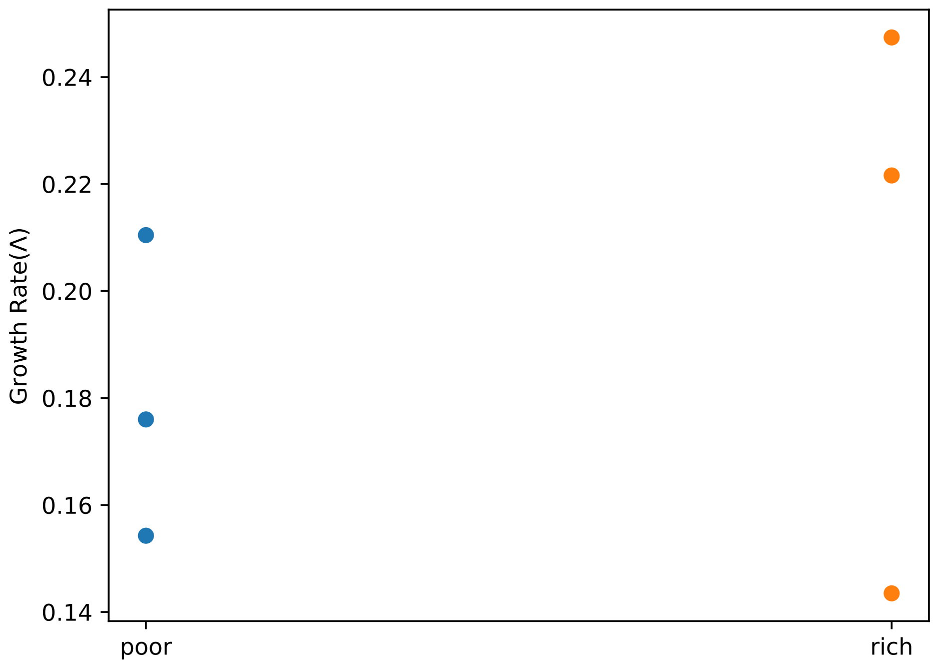

Supplementary Figure 10: Growth rates under control and glucose-deficient conditions
Growth rates (Λ) of *E. coli* populations under control and glucose-deficient conditions calculated according to the generalized Euler–Lotka equation are shown. Growth rates did not differ significantly between conditions (Mann–Whitney U test, $P = 0.33126 > 0.05$).

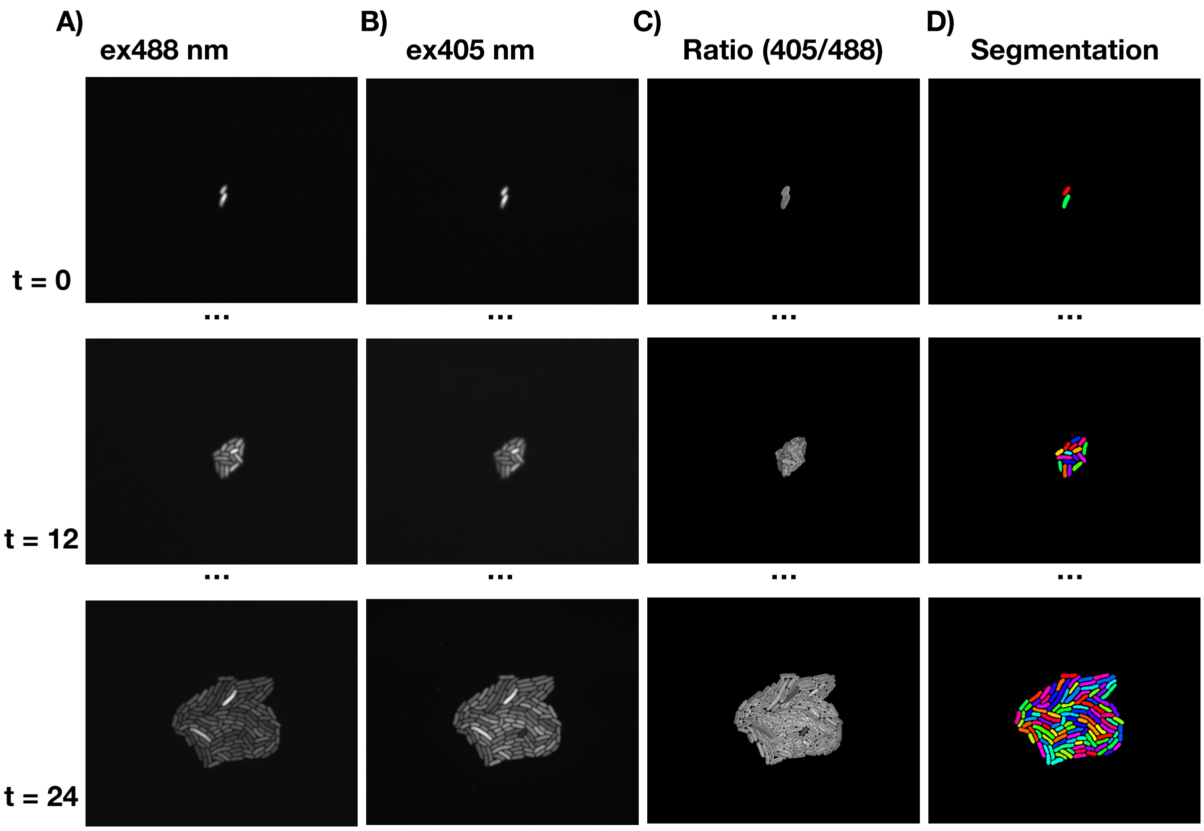

Supplementary Figure 11: Representative images used for data analysis Images of the *E. coli* populations obtained by using a custom microfluidics device and intermediate images created for data analysis. Images show a time slice of t = 0, 12, or 24 hours within the first sample of the control experiment. The images were obtained A) at 405 nm excitation, B) at 488 nm excitation, C) the ratiometric image obtained by dividing the intensity of the 405-nm image by that of the 488-nm image, and D) the segmentation image obtained by using the 405-nm excitation image and Schnitzcells [1] software. Each image was filtered with the median filter, and a rolling-ball algorithm was applied to remove noise. The analyses were done by using Scikit-Image [2]. The ratiometric and segmentation images were used to calculate the location and ATP concentration within each cell. Schnitzcells [1] was then used to track cellular lineages to create cellular lineage trees.

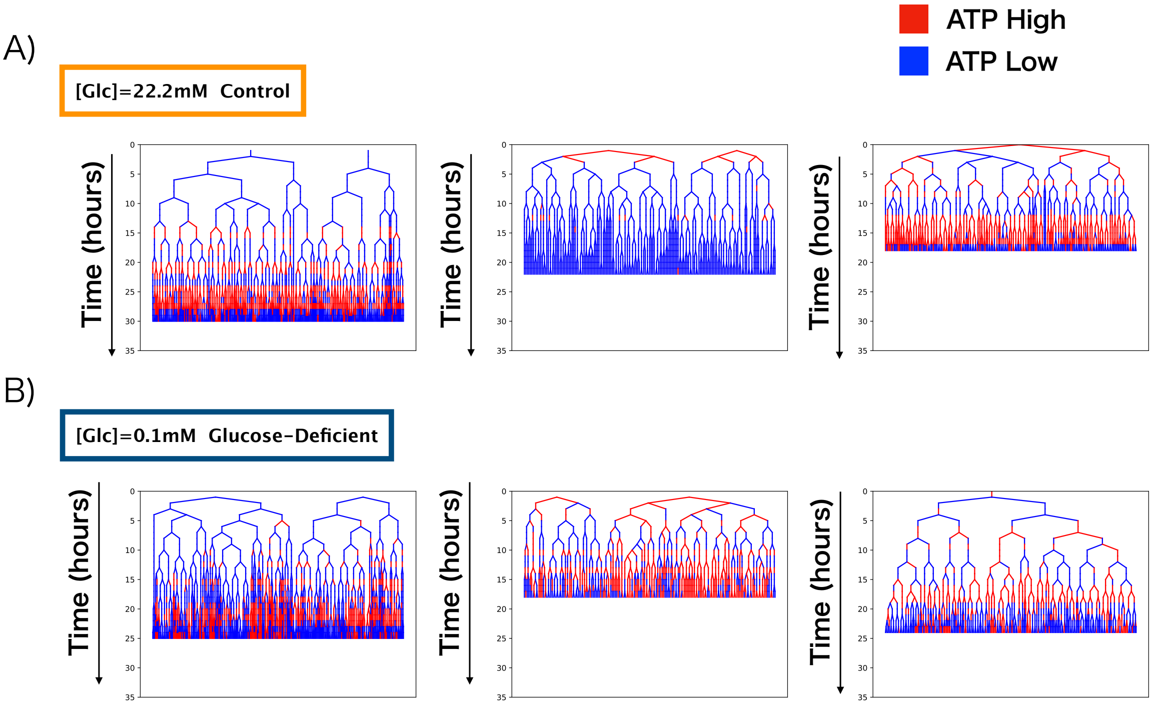

Supplementary Figure 12: State-transition lineages
The cellular lineages learned through Hidden Markov models (HMMs) for the control and glucose-deficient conditions. Cellular lineages obtained under (A) control conditions and (B) glucose-deficient conditions were split into two classes based on a predetermined threshold (n = 3 for each). The cellular lineages are color-coded (blue, low intracellular ATP; red, high intracellular ATP).

Supplementary Table 1: Various quantitative information in each *E. coli* lineages in Fig. 2 of the main body.

	Average Number of Divisions	Growth Rate (1/h)	Average ATP (mM)	Experimental Time (h)
Control-1 (left panel of Fig 2A)	6.7	0.143	3.426	30
Control-2 (middle panel of Fig 2A)	5.9	0.222	1.939	22
Control-3 (right panel of Fig 2A)	7.2	0.247	4.002	19
Control Averages	6.60	0.2044	3.1225	23.7
Control Variances	0.39	0.0029	1.1337	32.3
Deficient-1 (left panel of Fig 2B)	6.8	0.154	3.073	25
Deficient-2 (middle panel of Fig 2B)	6.0	0.176	3.790	18
Deficient-3 (right panel of Fig 2B)	7.0	0.210	3.446	25
Deficient Averages	6.62	0.1802	3.4364	22.7
Deficient Variances	0.28	0.0008	0.1284	16.3

Supplementary Table 2: P values of Mann–Whitney test comparing maximum amplitude and frequency between ATP classes under control and glucose-deficient conditions.

	P	
Wave property	Control	Glucose-deficient
Frequency (Hz)	3.91×10^{-4}	5.24×10^{-1}
Amplitude (F(k))	1.13×10^{-4}	1.43×10^{-1}

References

[1] Jonathan W Young, James C.W. Locke, Alphan Altinok, Nitzan Rosenfeld, Tigran Bacarian, Peter S Swain, Eric Mjolsness, and Michael B Elowitz. Measuring single-cell gene expression dynamics in bacteria using fluorescence time-lapse microscopy. *Nature Protocols*, 7(1):80–88, 2012.

[2] Stéfan van der Walt, Johannes L. Schönberger, Juan Nunez-Iglesias, François Boulogne, Joshua D. Warner, Neil Yager, Emmanuelle Gouillart, Tony Yu, and the scikit-image contributors. scikit-image: image processing in Python. *PeerJ*, 2:e453, 6 2014.
